# Recent Developments in Tuberculous Meningitis Pathogenesis and Diagnostics

**DOI:** 10.12688/wellcomeopenres.15506.3

**Published:** 2021-01-28

**Authors:** Fiona V Cresswell, Angharad G. Davis, Kusum Sharma, Robindra Basu Roy, Ahmad Rizal Ganiem, Enock Kagimu, Regan Solomons, Robert J. Wilkinson, Nathan C Bahr, Nguyen Thuy Thuong Thuong, Tuberculous Meningitis International Research Consortium

**Affiliations:** 1 Clinical Research Department, London School of Hygiene and Tropical Medicine, London, WC1E 7HT, UK; 2 Research Department, Infectious Diseases Institute, Kampala, PO Box 22418, Uganda; 3 MRC-UVRI-London School of Hygiene and Tropical Medicine Uganda Research Unit, Entebbe, Uganda; 4 University College London, London, WC1E6BT, UK; 5 Francis Crick Institute, London, NW1 1AT, UK; 6 Department of Medicine, Institute of Infectious Diseases and Molecular Medicine, University of Cape Town, 7925, South Africa; 7 Department of Medical Microbiology, Post-graduate Department of Medical Education and Research, Chandigahr, India; 8 Department of Neurology, Hasan Sadikin Hospital, Faculty of Medicine. Universitas Padjadjaran, Bandung, Indonesia; 9 Department of Paediatrics and Child Health, Faculty of Medicine and Health Sciences, Stellenbosch University, South Africa; 10 Department of Infectious Diseases, Imperial College, London, W2 1PG, UK; 11 Division of Infectious Diseases. Department of Medicine., University of Kansas, Kansas City, USA; 12 Oxford University Clinical Research Unit, Ho Chi Minh City, Vietnam

**Keywords:** Tuberculous meningitis, TBM, TB, HIV, pathogenesis, diagnostics

## Abstract

The pathogenesis of Tuberculous meningitis (TBM) is poorly understood, but contemporary molecular biology technologies have allowed for recent improvements in our understanding of TBM. For instance, neutrophils appear to play a significant role in the immunopathogenesis of TBM, and either a paucity or an excess of inflammation can be detrimental in TBM. Further, severity of HIV-associated immunosuppression is an important determinant of inflammatory response; patients with the advanced immunosuppression (CD4+ T-cell count of <150 cells/μL) having higher CSF neutrophils, greater CSF cytokine concentrations and higher mortality than those with CD4+ T-cell counts > 150 cells/μL. Host genetics may also influence outcomes with LT4AH genotype predicting inflammatory phenotype, steroid responsiveness and survival in Vietnamese adults with TBM. Whist in Indonesia, CSF tryptophan level was a predictor of survival, suggesting tryptophan metabolism may be important in TBM pathogenesis. These varying responses mean that we must consider whether a “one-size-fits-all” approach to anti-bacillary or immunomodulatory treatment in TBM is truly the best way forward. Of course, to allow for proper treatment, early and rapid diagnosis of TBM must occur. Diagnosis has always been a challenge but the field of TB diagnosis is evolving, with sensitivities of at least 70% now possible in less than two hours with GeneXpert MTB/Rif Ultra. In addition, advanced molecular techniques such as CRISPR-MTB and metagenomic next generation sequencing may hold promise for TBM diagnosis. Host-based biomarkers and signatures are being further evaluated in childhood and adult TBM as adjunctive biomarkers as even with improved molecular assays, cases are still missed. A better grasp of host and pathogen behaviour may lead to improved diagnostics, targeted immunotherapy, and possibly biomarker-based, patient-specific treatment regimens.

## Introduction

The pathogenesis of Tuberculous meningitis (TBM) is poorly understood. Mechanisms by which *Mycobacteria* disseminate from lung to the brain, key factors driving a dysregulated host response, and the pathogen specific factors influencing presentation and severity, compared to other forms of TB, are not well described. In recent years application of contemporary molecular biology ‘omics’ techniques to clinical samples, greater availability of advanced neuroradiology, emphasis on immune-mediated contributions to pathology, and use of refined experimental models of TBM have better illuminated its pathogenesis. A better grasp of these processes may also lead to improved diagnostics, targeted immunotherapy as well as a biomarker-based, patient-specific approach to personalized treatment. Diagnosis has been traditionally insensitive (AFB smear) and slow (culture). This has improved with the addition of GeneXpert MTB/Rif (Xpert) which gave sensitivities similar to culture in 2 hours (versus 2–4 weeks with culture). Subsequently, GeneXpert MTB/Rif Ultra (Ultra), a re-engineered version, has shown better sensitivities than culture in some settings. Yet, none of these technologies has adequate negative predictive value to ‘rule-out’ TBM. In this article we review important recently published studies that have informed our current understanding of TBM pathogenesis and diagnostics. We do not seek to present a comprehensive review of the history of TBM pathogenesis and diagnostics as a number of detailed papers have addressed this recently ^1– 3^. Rather we provide a commentary of key studies published within the last 5 years and summarise knowledge gaps and future considerations to enable progress in the field.

## TBM pathogenesis

### Dissemination to the central nervous system

Understanding of the microbial and immune processes that allow *M. tuberculosis* to disseminate from the respiratory epithelium to reach the meninges remains incomplete ^2, 4^. The foundations of what is known were laid through natural history and autopsy studies in the pre-chemotherapy era. The necessary steps to develop TBM include the pathogen surviving its initial encounter with the innate immune system at the respiratory epithelium and establishment of primary infection in the lung parenchyma with characteristic granulomatous inflammation ^5– 7^. Spread beyond the lungs likely occurs through the blood and may be preceded by local invasion to the lymphatic system. Donald and Schoeman have highlighted the possibility of coincident miliary TB in cases of TBM, particularly in young children, where tubercles of different sizes and ages have been described on the meninges and confirmed by magnetic resonance imaging (MRI) ^8, 9^. In children, miliary TB and TBM develop most often within 3 months of primary infection, when fresh anatomical changes are still found in the primary lung focus ^10^. In addition to children, people living with HIV (PLWHIV) are another vulnerable group who may be unable to control the infection in the lungs and therefore at risk of coincident miliary TB and TBM secondary to haematogenous dissemination of *M.tb* ^8, 11^. The contemporaneous nature of TBM and miliary TB illustrates that the “Rich focus” model (of a single meningeal/sub-cortical granuloma rupturing years after initial haematogenous dissemination discharging acid-fast bacilli into the sub-arachnoid space) ^12^ does not apply to all TBM cases and there may be more than one pathway to the development of meningitis following *M. tuberculosis* infection.

### Host immune response to TB infection in the CNS

**Figure 1.**
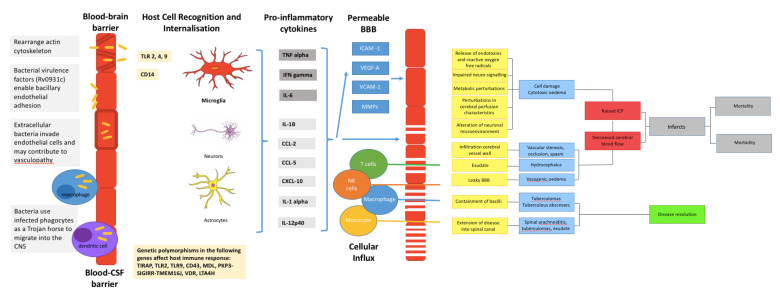
Illustrative summary of the pathogenesis of tuberculous meningitis (TBM). Reproduced with permission from author and Journal of Leukocyte Biology ^2^. A: *Mycobacterium tuberculosis* bacilli (M.tb) disseminate from the primary site of infection in the lung to seed the brain. The bacilli traverse the blood brain barrier (BBB) and blood cerebrospinal fluid barrier (BCSFB) through various virulence factors that enable the invasion of and migration through cerebral vascular endothelial cells, or are carried into the CNS by infected peripheral innate immune cells. B: In the CNS antigen recognition and internalization by microglia, neurons and astrocytes occurs, mediated by numerous host genetic factors. C: The resulting immune response stimulates the release of proinflammatory cytokines and chemokines and other immune mediators that contribute to the breakdown of the BBB and the influx of innate and adaptive immune cells from the periphery. D: A prolific inflammatory response ensues. The inflammatory exudate in the basal cisterns contributes to cerebral vascular pathology and the development of hydrocephalus and raised intracranial pressure. Vasogenic edema due to an influx of proteins through the leaky BBB, and cytotoxic edema as a result of cellular damage contribute to the raised pressure. The overall decrease in cerebral blood flow puts the brain at risk of ischemia, infarction and poor patient outcomes. In some cases the infection is controlled in discrete tuberculomas or abscesses, which may resolve with treatment and time.

The host immune response to TB bacilli in the sub-arachnoid space gives rise to a granulomatous inflammation predominantly affecting the basal meninges. Inflammatory exudates may obstruct the passage of cerebrospinal fluid (CSF), leading to hydrocephalus. Small and medium-sized intracerebral arteries can become inflamed and occluded, leading to cerebral infarcts. The majority of TBM pathology is believed to result from the host inflammatory response, which has been reviewed in depth elsewhere ^2^; several pro- and anti-inflammatory cytokines such as tumour necrosis factor-α (TNF-α), interferon-γ (IFN-γ), interleukin (IL) 1β, IL-6, IL-8, and IL-10 are shown to be induced in TBM ^13, 14^. Disequilibrium of pro- and anti-inflammatory cytokines influence the severity and course of TBM. Current understanding of key established mechanisms known to play a role in host immune response in TBM are summarised in Figure 1 ^2, 15^.

In the recent literature, the long-standing belief that excessive inflammation is the cause of death in TBM was brought into question by a recent immunopathogenesis study in Vietnam. In HIV-negative adults, associations between death and both *lower* CSF cytokines concentrations and lower CSF leucocyte counts (median 59 × 10 ^3^ cells/mL (IQR 13–240 × 10 ^3^ cells/mL) in those who died versus 135 × 10 ^3^ cells/mL (IQR, 48–298 × 10 ^3^ cells/mL) in survivors) were noted ^16^. These data support the notion that poor outcome from TBM, in the context of immunosuppressive treatment (adjunctive corticosteroids), is associated with an inadequate pretreatment inflammatory response in HIV-negative individuals. In a study of 120 Vietnamese adults with TBM included in a trial of adjunctive aspirin treatment, it was shown that there was an aspirin dose-dependent inhibition of thromboxane A _2_ and upregulation of pro-resolving CSF protectins, resulting in potential reduction in new infarcts and deaths by day 60 of treatment in microbiologically confirmed TBM patients ^17^. A further study investigated concentrations of host protective lipid mediators (specialized proresolving mediators, SPMs) in CSF. Prostaglandins and cysteinyl leukotrienes were found to be reduced in more severe cases, while the lipoxygenase 5-derived 13-series resolvin (RvT)2, RvT4, and 15-epi-lipoxin B4, were significantly increased in survivors. These data suggest SPMs may play an important role in TBM pathogenesis ^18^.

Among 608 Indonesian adults with suspected TBM, higher CSF and blood neutrophil counts (HR 1.10 (95%CI 1.04–1.16) per 10% increase and HR 1.06 per 10 ^9^ neutrophils/L increase; (95% CI 1.03–1.10), respectively) were associated with mortality ^19^. Flow-cytometry on blood in a subset of 160 HIV-negative adults with TBM showed lower αβT and γδT cells, NK cells and MAIT cells in TBM subjects compared to 26 pulmonary TB adults (2.4 to 4-fold, all p < 0.05) and 27 healthy controls (2.7-7.6-fold, p < 0.001), but higher neutrophils and classical monocytes (2.3 - 3.0-fold, p < 0.001). CSF flow cytometry of TBM patients showed a predominance of αβT and NK cells, associated with better survival, as well as the presence of MAIT cells, previously undescribed in CSF ^20^. Indonesian HIV-negative TBM patients showed a strong myeloid blood response and a remarkably broad lymphoid CSF response including innate lymphocytes, however there was little correlation between blood and CSF compartments ^20^.

These recent studies in Vietnamese and Indonesian adults with TBM, aimed at gaining insights into mechanisms of the inflammatory response in disease pathogenesis, used novel and high-resolution methods to look at lipid mediator profiles and immune cell populations. Data indicated specific lipid mediator signatures and cell populations that are associated with disease severity before treatment and mortality; these should be considered for host-directed therapy of TBM.

***Host genetic and metabolic factors***

**Figure 2.**
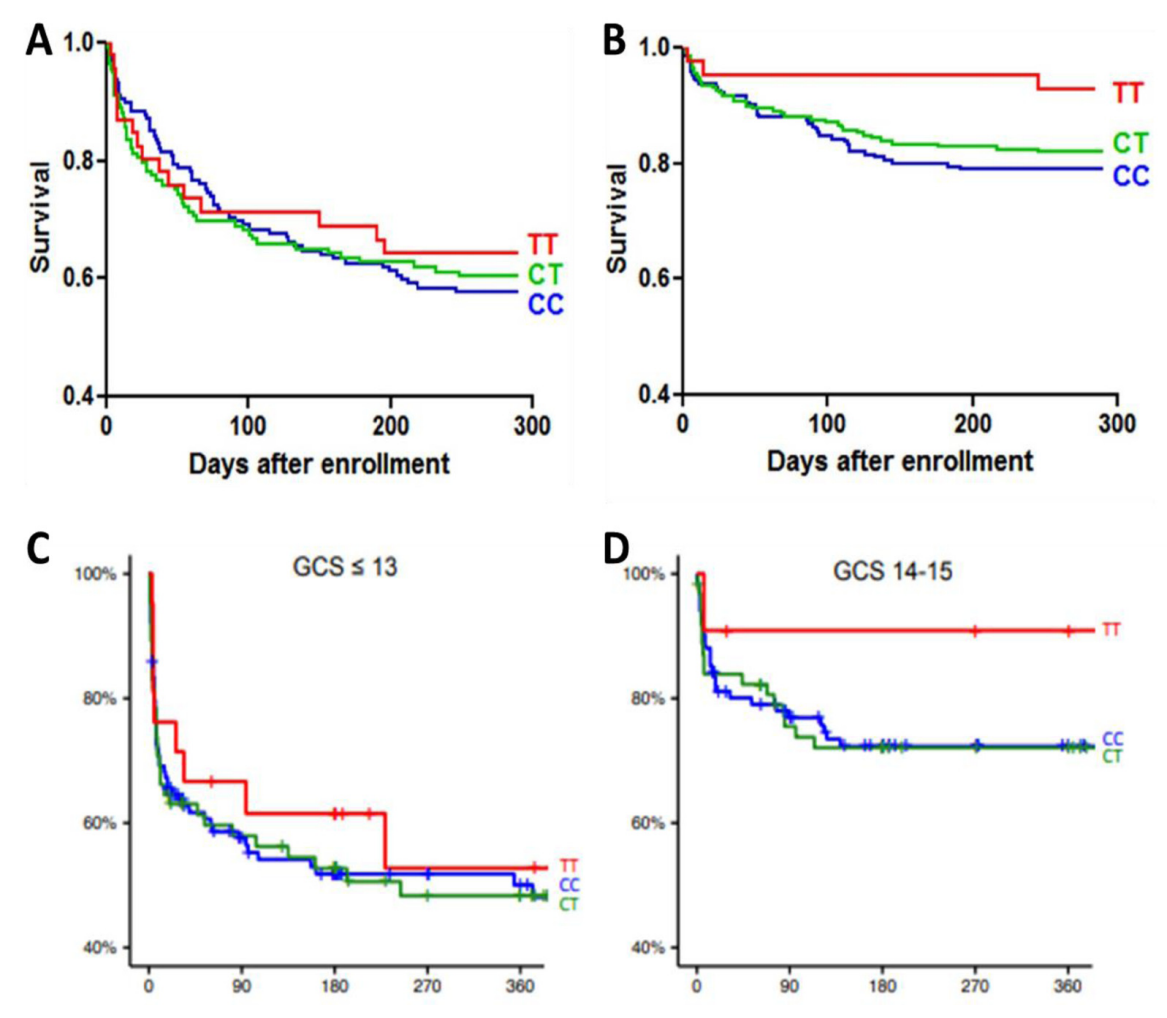
Kaplan-Meier survival curves stratified by LTA4H genotype. Figures **A** and **B** show survival in 763 patients with tuberculous meningitis in Vietnam ^16^, with human immunodeficiency virus (HIV) infection ( **A**), and without HIV infection ( **B**). In HIV-infected patients, case-fatality rates were 34.8% (16 of 46) in those with TT genotype, 42.1% (61 of 145) in CT genotype, and 38.8% (52 of 134) in CC genotype. In HIV-uninfected patients, case-fatality rates were 7.1% (3 of 42) in TT, 21.4% (40 of 187) in CT, and 18.7% (39 of 209) in CC. Figures **C** and **D** show survival in 375 patients with tuberculous meningitis in Indonesia ^19^. These patients are HIV-uninfected with severe (GCS ≤ 13) ( **C**) or milder (GCS 14–15) disease ( **D**). In a recessive model, TT genotype versus CT/TT combined had HR 0.81 (95% CI 0.41-1.62, p = .550) in severe and 0.31 (95% CI 0.04-2.25, p = .156) in milder disease.

More efficient and cost-effective genomics platforms have enabled of late better understanding of variable host responses in TBM through the study of host genetics. Polymorphisms in CD43 encoding a surface glycoprotein involved in *M.tb* adhesion and proinflammatory cytokine induction and PKP3-SIGIRR-TMEM16J gene region encoding a negative regulator of TLR/IL-1R signalling have both been linked to survival in TBM ^21, 22^. However, the greatest interest has been around the role of leukotriene A4 hydrolase (LTA4H). LTA4H catalyzes the final step in the synthesis of leukotriene B4 (LTB4), a potent chemoattractant and pro-inflammatory eicosanoid. A common functional promoter variant rs17525495 in the LTA4H gene can predict survival and dexamethasone responsiveness in HIV-uninfected adults with TBM ^16, 23^. This human candidate gene association study was guided by findings in a zebra fish model where LTA4H was found to determine the balance of pro-inflammatory and anti-inflammatory eicosanoids in response to mycobacterial infection ^16, 24^. In a retrospective study in Vietnamese HIV-uninfected adults with TBM, while *LTA4H rs17525495* TT and CC genotypes were both associated with susceptibility to mycobacterial infection, the associations involved opposing inflammatory states: high inflammation for the TT genotype and low inflammation for the CC genotype. CT genotype had an intermediate inflammatory response and were more likely to survive TBM. Dexamethasone treatment improved survival in TT genotype patients with hyper-inflammatory response but was possibly harmful to CC patients with hypo-inflammatory response ^23^. A later prospective study in Vietnam reported that in TBM HIV-uninfected adults, *LTA4H* genotype influences cytokine inflammatory response and correlates with TBM severity, Figure 2 ^16^. More importantly, this study confirmed that the *LTA4H* genotype determined corticosteroid responsiveness and survival. 

Interestingly, LTA4H genotype did not predict outcomes in Indonesian adults with TBM, but there was a trend towards improved survival with TT genotype compared to CC or CT genotype, Figure 2 ^19^. A clinical trial is currently underway in Vietnam (NCT03100786) to evaluate LTA4H genotype-directed corticosteroid therapy, an exciting example of personalised medicine in TBM ^25^.

Although prior studies have considered sodium, glucose and lactate as related to TBM pathogenesis, recent developments in the application of targeted metabolomics have provided greater insight in the role of tryptophan, a potential key metabolite in TBM. This amino acid required for protein biosynthesis is a precursor to serotonin and melatonin (serotonin pathway) and kynurenine and quinolinic acid (kynurenine pathway). The latter is stimulated at the expense of the former by pro-inflammatory cytokine such as IL-6, TNF-alpha and IFN-gamma via indoleamine 2, 3-dioxygenase. In a recent study of serum and CSF metabolites, low levels of tryptophan were associated with survival ^26^. One theory regarding this association could be the neuroprotective effects of the associated kynurenine pathway downstream metabolites. Either this pathway, or the 11 genetic foci related to CSF tryptophan metabolism could have novel clinical implications for TBM ^26^.

### HIV co-infection and immune reconstitution inflammatory syndrome

HIV infection is a strong independent predictor of death from TBM (hazard ratio, 3.94; 95% confidence interval (CI), 2.79–5.56) ^27^. The role of adjunctive corticosteroids in HIV-associated TBM is inconclusive (relative risk of death with adjunctive steroids, 0.78; 95% CI, 0.59 to 1.04; P=0.08) ^28^ and a randomized placebo-controlled trial is underway (NCT03092817) to address the use of steroids in HIV-associated TBM. Pathogenesis studies in PLWHIV are required to identify the unique pathogenic determinants of poor prognosis. Thuong *et al*. compared the pretreatment CSF cells and cytokine profiles of 764 HIV-positive and HIV-negative participants in Vietnamese TBM clinical trials. HIV-positive individuals had higher mean CSF neutrophil percentage (17% vs 5%; P < .0001) and global cytokine expression (aside from IL-10 which inhibits response to *M. tuberculosis*) than their HIV-negative counterparts. PLWHIV with CD4+ T-cell counts <150 cells/μL showed higher median CSF neutrophil percentage (25%), than those with a count ≥150 cells/μL (neutrophils 10%; P=0.021) and patients without HIV infection (neutrophils 5%; P<0.0001). Of patients with a CD4+ T-cell count of <150 cells/μL, 44% (105 of 238) died, compared with 13% (5 of 39) with a count of ≥150 cells/μL and 19% (83 of 439) without HIV infection ^16^. These findings, amongst others, suggest a role for neutrophils in the immunopathogenesis of HIV-associated TBM ^29^.

Marais *et al.* conducted longitudinal analyses of paired blood and CSF samples in South Africans with HIV-associated TBM, describing the relationships between the development of immune reconstitution inflammatory syndrome (IRIS) and CSF leucocytes, the concentrations of >30 blood and CSF inflammatory mediators, and blood transcriptional profiles. They found TBM-associated CNS IRIS to have an inflammatory signature characterized by neutrophil and inflammasome-mediated proinflammatory responses ^30, 31^. The neutrophil-dependent inflammatory activation could be detected in peripheral blood before the start of TB treatment and therefore has potential to predict who will develop IRIS.

***Brain Injury Markers***

The study of neurodegenerative-associated proteins to describe extent and type of brain injury post TBM has recently been explored through omics analysis, approaches which strive to understand genetic or molecular profiles of humans, particularly in paediatric TBM. In lumbar CSF of children with TBM, S100B and NSE (structural proteins of the CNS, and biomarkers of CNS tissue damage) at disease onset were associated with poor outcome, as was highest concentration overall and an increasing profile over time in S100B, NSE, and GFAP neuromarker concentrations increased over time in those who died (whilst inflammatory markers decreased), and were overall highest in those with cerebral infarction ^32^. It is of interest that despite markers of inflammation reducing, proteins traditionally associated with neurodegenerative processes continued to rise.

In ventricular CSF of children with TBM, transcriptome analysis has revealed significant enrichment of transcripts associated with neuro-excititoxicity predominantly driven by glutamate release and NMDA binding and receptor uptake ^33^. Upregulation of genes associated with nitric oxide, cytochrome c, brain injury proteins like myelin basic protein, and proteins including tau, amyloid-beta and apo- lipoprotein were also seen ^33^; many of which have also been described in in neurodegenerative conditions such as Alzhiemer’s disease and traumatic brain injury ^34^.

These findings raise the possibility of ongoing brain injury which in TBM seem to occur following ischaemic injury, despite resolving acute inflammation ^32^. Further studies, including those which investigate the longer-term pathogenic processes in TBM are required to validate these results and understand further neurological sequelae including those which may indicate a post-infectious process in TBM.

### Neuroimaging in pathogenesis studies

Technical advances and increasing availability of imaging modalities have recently enabled research in which imaging is used to assess pathogenic mechanisms in TBM *in vivo* in animal and human subjects. In a blood and CSF biomarker study of childhood TBM tuberculomas, magnetic resonance imaging has been used to note an association between tuberculomas and elevated interleukin (IL) 12p40, interferon-inducible protein 10, and monocyte chemoattractant protein 1 concentrations, whereas infarcts were associated with elevated TNF- α, macrophage inflammatory protein 1α, IL-6, and IL-8 ^32^. ** Specific sequences can also be used to describe morphology of structural damage and correlate this to meaningful clinical measures. For instance poorer Diffusion Tensor Imaging (DTI) parameters of white matter integrity in the anterior cingulate gyrus, parahippocampal gyrus and globus pallidus are associated with worse neuropsychological performance ^35^. A further study by the same group used Diffeomorphic Anatomical Registration Through Exponentiated Lie Algebra (DARTEL) voxel-based morphometry (VBM) to assess the integrity of grey matter in these same TBM patients ^36^. Patients with TBM performed significantly poorer on the digit symbol, similarities, block design, matrix reasoning, and letter-number sequencing subtests of the Wechsler Adult Intelligence Scale compared to healthy adults. These changes correlated with smaller grey matter volumes in the right thalamus, right superior temporal gyrus, right precuneus, right middle temporal gyrus, left putamen, right caudate nucleus, and right middle temporal gyrus ^36^. These studies suggest that structural damage can be cortical as well as subcortical which may in turn be related to degree of long-term impairment. This has implications for understanding long term outcomes particularly neurocognitive impairment in TBM, which in light of these findings may share features with other forms of dementia (including vascular and HIV associated neurocognitive impairment) where a subcortical pattern of neurocognitive impairment (including frontal and executive functions) can be observed.

A rabbit model study of childhood TBM, utilized ionized calcium binding adapter molecule (Iba-1) to approximate microglial activation with flurodeoxyglucose-positron emission tomography (FDG-PET) and demonstrated the presence of activated microglia and macrophages localized to TB lesions ^37^. In humans, case reports and a prospective study have advocated the use of FDG-PET as a diagnostic tool, as it has been effective in detecting extra-cranial evidence supportive of a TBM diagnosis ^38– 40^. The role of FDG-PET in unravelling time course of inflammation in TBM remains to be seen, although it has played a role in understanding Alzheimer’s, a disease in which, similar to TBM, inflammation plays a key pathogenic role ^41, 42^.

### Pathogen factors: bacillary load, pathogen strain and virulence factors

TBM patients generally have low bacterial loads in CSF which causes difficulties in both diagnosis and ability to study bacterial load evolution-related pathophysiology. The time-to-positivity of a culture and cycle threshold (Ct) of nucleic acid amplification tests such as GeneXpert MTB/Rif (Xpert) can provide an indication of likely bacterial burden ^43^. Over 50% of diagnosed cases are microbiologically undetectable and defined as ‘probable’ or ‘possible’ TBM which obviously limits this approach ^44^. Marais *et al*. showed that in patients where *M.tb* was cultured from CSF taken before and after two weeks of anti-tuberculosis treatment, there was a 9.3-fold increased risk of subsequently developing TBM-IRIS, although the sample size is small with 15 TBM-IRIS patients compared with 6 non-TBM-IRIS patients ^45^. Thuong *et al*. found that among 692 Vietnamese adults with TBM, pre-treatment CSF *M.tb* load (by Xpert Ct) was correlated with increased CSF neutrophil counts, increased cytokine production, and new neurological events after treatment initiation, but not death ^43^.

In addition, epidemiological trends of *M.tb* lineage from TBM (n=73) and pulmonary TB (n=220) patients in Thailand showed that the Indo-Oceanic lineage is more frequently found in TBM patients (41% versus 13% in PTB) ^46^. This association did not hold true in Indonesia, though specific genetic variations were identified which were associated with TB phenotype, including one (Rv0218) whose encoded protein may play a role in host-pathogen interaction ^47^.

### Host-pathogen interactions

**Figure 3.**
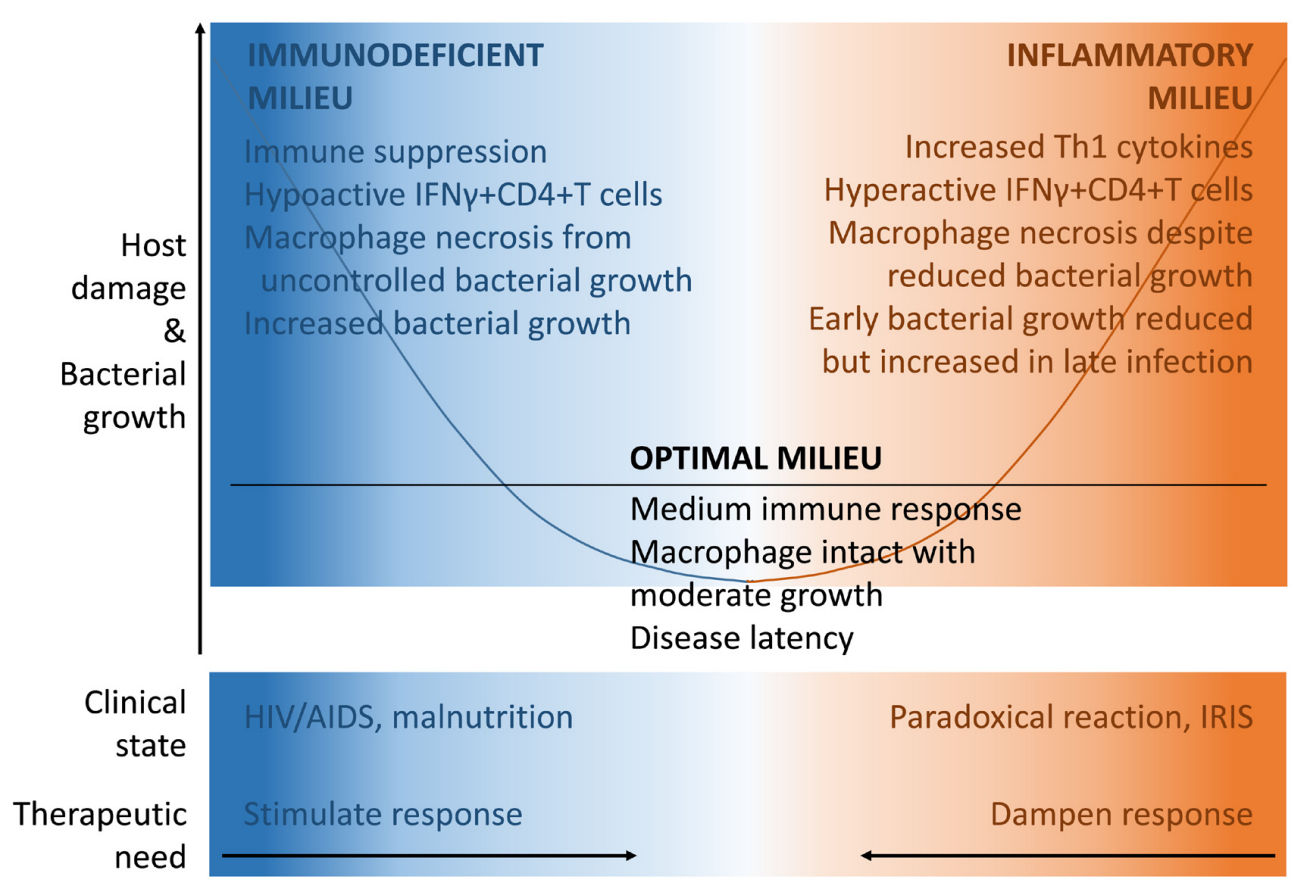
Outcomes of the host – *M. tuberculosis* interaction depicted by the basic parabola of the damage-response framework. On the left side of the parabola, shaded in blue, the immune system fails to limit mycobacterial growth and invasion which results in host damage. On the right side, shaded in red, the immune response is excessive and the resultant inflammation and host-damage. The proportion of the parabola lying below the black line represents disease latency, which is not associated with clinically evident host damage. On the blue side therapeutic interventions could be targeted at stimulating an immune response, whilst on the red side therapeutic interventions could aim to dampen immune response.

It is estimated that the global burden of latent TB infection (LTBI) is approximately 23.0% (95% CI 20.4%–26.4%), amounting to approximately 1.7 billion people ^48^. Innate immune responses are critical to control TB infection yet also contribute to tissue damage. This delicate balance is illustrated in the damage response framework which provides a theory of microbial pathogenesis that incorporates the contributions of both host and microbe to host damage that stems from host-microbe interaction ^49, 50^. This framework likely applies to TBM based on evidence of both failed immunity and excessive inflammation being linked to increased TBM pathology, see Figure 3 ^23, 51, 52^. Both the microbe and the host contribute to host damage and where an individual patient’s immune response lies on the continuum of the damage response framework parabola determines the nature of the disease process ^16, 43, 53^. Evidence from recent studies shows *LTA4H* genotype, CSF cytokines and CSF immune cells such as neutrophils are determinants of inflammatory state, which impacts both bacterial growth and host damage and thus leads to different outcomes. The current one-size-fits-all approach to TBM treatment fails to recognize divergent pathologies and may explain the poor outcomes in certain populations. Being able to identify where on the parabola an individual lies and tailoring therapy to achieve the optimal milieu is an approach that warrants further investigation. *LTA4H* genotype is an example of using host genotype to predict inflammatory response and to tailor treatment by host directed therapy. Omics technology are now being used to identify additional host genetic markers and treatment targets in TBM.

## TBM diagnostics

The field of TBM diagnostics has evolved rapidly in recent years with both complex and low-tech assays being explored in a variety of populations. Whilst progress has been made, no single assay can be used as a rule out test. The characteristics of CSF tests studied to date are summarized in Table 1.

**Table 1.** Summary of TBM CSF Diagnostic Test Performance and Limitations.

Test	Sensitivity *	Specificity *	Time to Results	Strengths	Limitations
**AFB Smear**	10–34%	95–100%	Hours	Rapid, cheap, widely available, specificity	Poor sensitivity in most settings
**Culture**	48–60%	100%	2–6 weeks	Sensitivity, excellent specificity, antimicrobial resistance testing	Slow, lab infrastructure, costly, inadequate NPV
**Adenosine** **deaminase**	89%	91%	Days	Good sensitivity, low CSF volume requirement	Cost, lab infrastructure, false positives, study heterogeneity, variable test performance
**IGRA**	77%	88%	Days	Sensitivity	Cost, lab infrastructure, false positives, indeterminate results, varied study designs and cut-points
**Antibodies**	75–91%	91–98%	Days	Sensitivity	Variable study design, lack of commercial assays, false positives, numerous different targets
**IL-13, VEGF,** **cathelicidin** **LL-37 ^a^**	52%	95%	Days	Sensitivity, low CSF volume requirement	Cost, lab infrastructure, requires validation, technical expertise
**IFNg, MPO,** **VEGF ^a^**	91%	100%	Days	Sensitivity, low CSF volume requirement	Cost, lab infrastructure, requires validation, technical expertise
**Traditional** **NAAT**	68–82%	100%	Days	Sensitivity, specificity	Cost, lab infrastructure, many are ‘in-house’ tests, variable study design, variable targets, stringent operational conditions, technical expertise
**Xpert MTB/** **Rif**	40–70%	98–100%	Hours	Sensitivity, specificity, rapid, ease of use, detects rifampin resistance, widely distributed platform	Cost, inadequate NPV, variable study design and performance
**Xpert Ultra**	47–95%	100%	Hours	Sensitivity, specificity, rapid, ease of use, detects rifampin resistance, widely distributed platform	Cost, inadequate NPV, variable study design and performance
**CRISPR-MTB**	73%	98%	Hours	Low CSF volume, sensitivity, specificity, isothermal	Cost, lab infrastructure, stringent operational use, technical expertise, requires validation
**mNGS**	67%	98–100%	Days- Weeks	Can detect alternative pathogens, sensitivity, specificity	Cost, lab infrastructure, very stringent operational use, technical expertise, requires validation
**Alere TB LAM**	22–24%	95%	Minutes	Rapid, cost, heat stability, limited lab expertise or infrastructure requirements	Sensitivity, intra-operative variability, inadequate NPV
**Fujifilm** **SILVAMP** **TB LAM**	52–74%	98%	1 hour	Rapid, sensitivity, heat stability, limited lab expertise or infrastructure requirements	Cost, intra-operative variability, inadequate NPV

*All sensitivity and specificity values are approximate, based on current literature with the understanding that variability occurs between studies and with local disease prevalence. ^a^: Reported values are for paediatric patients only TBM: Tuberculous meningitis; AFB: acid-fast bacilli; NPV: negative predictive value; IGRA: interferon gamma release assay; IL: interleukin; VEGF: vasoactive endothelial growth factor; IFNg: interferon gamma; MPO: myeloperoxidase; NAAT: nucleic acid amplification tests; Xpert: GeneXpert MTB/Rif; Xpert Ultra: GeneXpert MTB/Rif Ultra; CRISPR-MTB: Clustered regularly interspaced palindromic repeat associated proteins – *Mycobacteria tuberculosis*; mNGS: metagenomic next generation sequencing; LAM: lipoarabinomannan;

### Host-based diagnostic biomarkers

**Figure 4.**
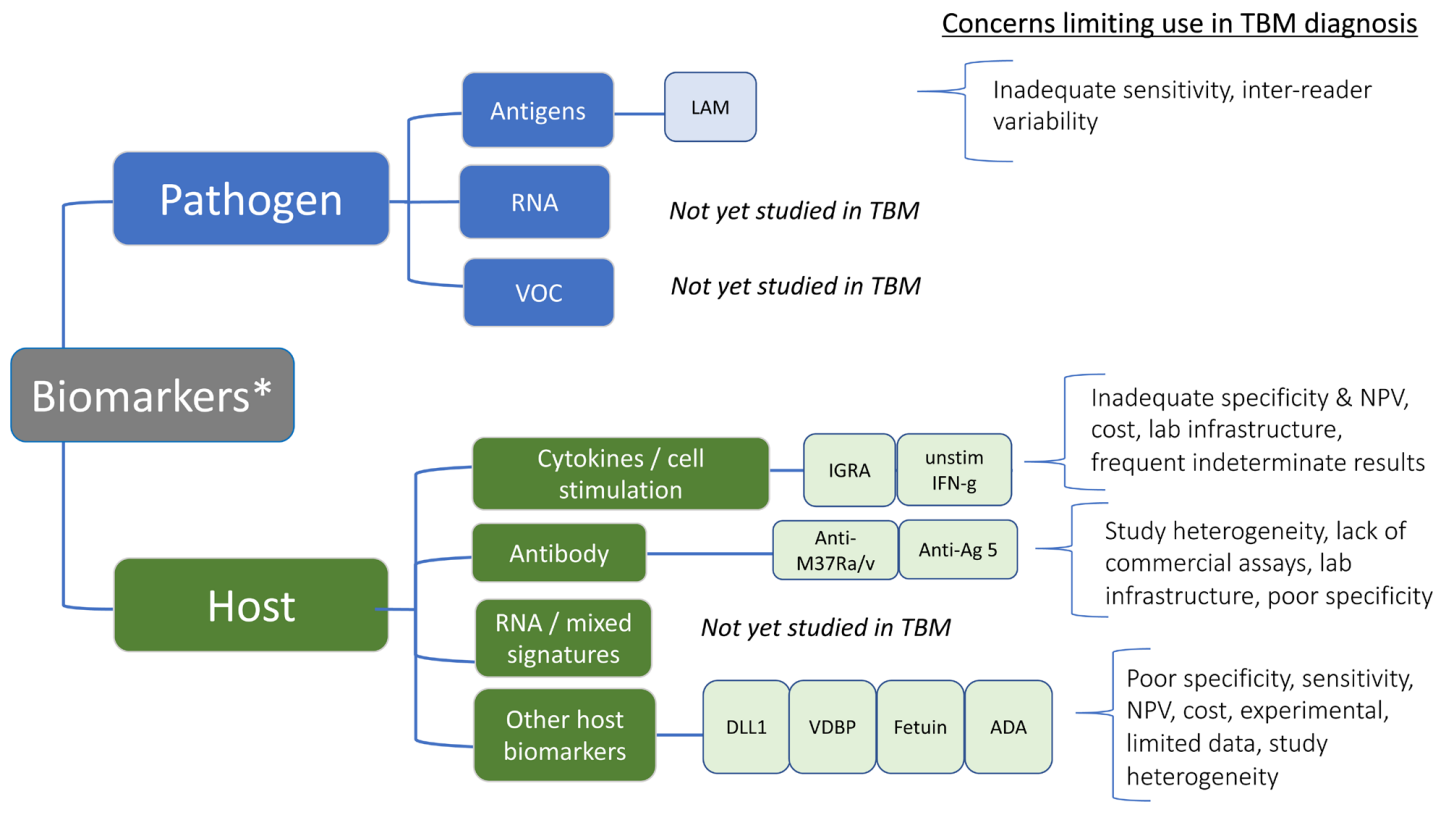
Novel Host and Pathogen biomarkers for diagnosis of tuberculous meningitis ^54– 60^. LAM = lipoarabinomannan, RNA = riboneucleic acid, VOC = volatile organic chemicals, IGRA = inferferon gamma resease assay, DLL1 = delta-like ligand 1, VDBP = vitamin d binding protein, ADA = adenosine deaminase. * This image focuses only on novel host or TB biomarkers for diagnosis of TBM and does not incorporate traditional tools such as culture or AFB smear, or newer nucleic acid amplification tests in use commonly such as GeneXpert or GeneXpert Ultra, or experimental techniques such as metagenomic next generation sequencing.

Traditional diagnostic techniques for TBM include CSF smear microscopy for acid fast bacilli (rapid and cheap but insensitive in most settings, 10–15%) and CSF culture (improved sensitivity of 50–60% but results in 2–6 weeks with a biosafety lab level three requirement) ^3^. Given the limitations of traditional, diagnostic tests for TBM that focus on bacillary detection, there is interest in the utilization of host-based diagnostic biomarkers for diagnosis of TBM, Figure 4. Adenosine deaminase (ADA), produced by lymphocytes, is an important regulator of follicular helper T-cells. ADA is commonly used for diagnosis of TB from other, typically extra-pulmonary locations and numerous studies have considered ADA for diagnosis of TBM ^54^. One 2017 meta-analysis found ADA to have a pooled sensitivity of 89% (95% CI 84–92%) with pooled specificity 91% (95% CI, 87–93%) ^54^. Yet ADA use for TBM diagnosis has been limited by the high cost of the test, required sophisticated lab infrastructure, study heterogeneity, inadequate negative predictive values, and variable test performance.

A number of studies have considered unstimulated CSF interferon gamma (IFN-γ) levels as a diagnostic test, in general, a high number of false positive results has limited the utility of CSF IFN-γ ^55, 56^. For instance, in one study of 39 controls (n=12 viral, n=16 purulent, n=11 cryptococcal meningitis) and 30 subjects with TBM while median IFN-γ levels were higher amongst subjects with TBM, diagnostic accuracy was inadequate ^56^. At the strongest cut-point (81pg/mL) determined by receiver operator curve analysis, positive predictive value was only 81% with positive results occurring in 2/12 (17%) with viral meningitis, 3/16 (19%) with purulent meningitis, and 1/11(9%) with cryptococcal meningitis ^56^.

Interferon gamma release assays (IGRAs) are commonly used to infer LTBI. A 2016 meta-analysis of six studies performing CSF IGRA’s found a pooled sensitivity and specificity of 77% (95% CI 69%-84%) and 88% (95% CI 74%-95%), respectively, for TB meningitis, though reference standards varied by study ^57^. Limitations of IGRA include high cost, the need for advanced lab infrastructure, frequent "indeterminate” results, and false positives associated with other causes of meningitis. Additional host biomarkers including delta-like ligand 1, vitamin D binding protein, and fetuin have been evaluated in CSF though none were found to have satisfactory performance ^58^. Numerous CSF antibodies to *M.tb* in CSF has also been evaluated. Huang and colleagues found pooled sensitivities of 91% (95% CI 71–98%) for anti-M37Ra across five studies, 84% (95% CI 71–92%) for anti-antigen-5 across eight studies, and 84% (95% CI 71–92%) across 12 studies for anti-M37Rv, again using a variety of reference standards (making the pooled estimates somewhat flawed) ^59^. Use of blood antibody assays are discouraged for the diagnosis of TB, and their utility in CSF is limited by heterogeneity and the lack of a uniform reference standard across research studies as well as a lack of commercial assays.

Though on the surface, many of these markers look to have promise, uptake has been limited. Heterogeneity in study design and widely variable study performance has limited the consensus regarding the utilities for most host-based tests. Further, many of these tests require sophisticated laboratory infrastructure, are costly, and in some cases are not commercially available. None of these CSF tests are routinely used and as far as the authors are aware, none are actively being studied further. 

In the field of pulmonary tuberculosis, host RNA transcriptomic signatures have been leveraged to predict incipient and active tuberculosis with moderate short-term (<3 month) sensitivity (41–81%) ^61^. A whole blood 3-gene signature (GBP5, DUSP3, KLF2) has been shown to accurately differentiate active pulmonary tuberculosis from healthy controls (area under receiver operator curve, AUC 0.90), latent TB infection (AUC 0.88) and other diseases ^62^ (AUC 0.84). Whether this 3-gene signature in whole blood has diagnostic value in TBM, whereby the inflammation can be compartmentalised to the CNS, remains to be investigated. An ongoing study in Uganda is adopting a machine learning approach to develop a classifier that categorises patients as TBM or not-TBM based on their CSF RNA signature. In CSF, genes predominantly predicted TBM (FTL, NFKBIA, SOD2, GBP5) and the classifier demonstrated good sensitivity and specificity (unpublished data).

### Biomarkers in children

The often-dismal outcome of TBM is contributed to by delayed diagnosis and/or initiation of treatment, especially in high burden settings ^4^. Currently available diagnostic test performance is especially poor in young children with TBM. Thus, diagnosis of childhood TBM is mostly based on a combination of clinical findings, CSF analysis and radiological findings ^63^. Even so, there are often multiple missed opportunities prior to a diagnosis of childhood TBM ^64^. Since it can be challenging to identify bacilli in paediatric extrapulmonary TB, the use of host or pathogen biomarkers to aid diagnosis is being explored. Host biomarker-based tests have shown promise in extrapulmonary TB outside of the CNS and therefore have potential applications in TBM ^65^. Recent technological advances have made it possible to screen for many biomarkers in as little as 3 µl of sample using the Luminex multiplex cytokine beaded arrays, albeit in research context currently, rather than routine clinical practice.

In a study evaluating disease-specific CSF biomarkers of paediatric TBM, a combination of 28 cytokines and soluble mediators were assessed; 27 host biomarkers by Luminex multiplex bead array technology (Bio-Rad Laboratories) and cathelicidin LL-37 concentration by using an enzyme-linked immunosorbent assay (ELISA) kit (USCN Life Science). A three-marker CSF biosignature comprising IL-13, VEGF and cathelicidin LL-37, diagnosed childhood TBM with a sensitivity of 52%, specificity of 95%, with positive and negative predictive values of 91% and 66% respectively. Cut-off values for VEGF, IL-13 and cathelicidin LL-37 were 42.92 pg/mL, 37.26 pg/mL and 3221.01 pg/mL respectively ^15^. Further evaluation of this three-marker CSF biosignature in a different cohort revealed positive and negative predictive values of 90% and 59.5% respectively, however with different cut-off values for VEGF, IL-13 and cathelicidin LL-37 of 9.4 pg/ml, 524.9 pg/ml and optical density of 0.045 respectively ^66^. In this study investigating 69 potentially-useful host biomarkers for childhood TBM (23 children with TBM and 24 controls) comprising a combination of cytokines and soluble mediators in CSF (cathelicidin LL-37 by using an ELISA kit purchased from Elabscience Biotechnology Inc. (catalog #E-EL-H2438) and the rest by Luminex assay), 28 proteins including IFN-γ, TNF-α, MPO, MMP-8, MMP-9, MIP-4 and CXCL9 amongst others, when analysed individually, showed areas under the receiver-operating curve (AUC) ≥0.80. When combined, biomarkers IFN-γ, MPO and VEGF showed good accuracy (AUC = 0.97, up to 91.3% sensitivity and up to 100% specificity), as well as ICAM-1, MPO, CXCL8, and IFN-γ (AUC of 0.97, up to 87.0% sensitivity and up to 95.8% specificity). Cut-off values for VEGF, IFN-γ, MPO, ICAM-1 and CXCL8 were >9.4 pg/ml, >99.5 pg/ml, >25823.0 pg/ml, >1372.0 pg/ml and >394.8 pg/ml, respectively ^66^.

Limitations of the studies assessing CSF host biomarkers in childhood TBM include relatively small sample size, and therefore few children with confirmed TBM, confirmed meningitis due to other pathogens, and confirmed HIV co-infection. External validation is a necessity in order to generalize the clinical usefulness of the prediction model in an independent group of patients. the potential of CSF-based biosignatures, a further limitation is the invasive nature of CSF collection, and blood or urine-based inflammatory biosignatures require exploration. In a study evaluating serum biomarkers, the combination of CRP, IFN-γ, IP-10, CFH, Apo-A1 and SAA showed moderate diagnostic accuracy for clinically-defined TBM, including both ‘definite’ and ‘probable’ TBM (AUC of 0.75, sensitivity of 69.6% and specificity of 62.5%). A three-biomarker combination of adipsin, Aβ42 and IL-10 showed improved accuracy (AUC of 0.84, sensitivity of 82.6% and specificity of 75.0%). Cut-off values for CRP, IFN-γ, IP-10, CFH, Apo-A1, SAA, adipsin, Aβ42 and IL-10 were >80721.0 ng/ml, <61.5 pg/ml, <57.2 pg/ml, >350185.0 ng/ml, >287512.0 ng/ml, >59894.0 ng/ml, <2393.0 ng/ml, <278.4 pg/ml and <7.0 pg/ml, respectively. Although sample size was small, these biomarkers warrant further exploration ^67^.

### Pathogen-based diagnostics

The absence of a perfect gold standard for use in TBM diagnostic studies means that the results must be interpreted with an awareness of the pros and cons of the reference standard used. The 2010 uniform TBM case definition which defines cases as ‘definite’, ‘probable’, ‘possible’ or ‘not TBM’ is the most standardised tool to use when defining a case definition ^63^. This case definition was derived by expert consensus rather than being data-driven and, although designed to be applicable to any age, HIV infection status or geographical setting, may perform better in some contexts than others. In HIV-negative populations a reference standard of ‘definite, probable or possible’ is often used, however in PLWHIV including ‘possible’ in the reference standard can be imprecise due to the wide variety of infectious and non-infectious aetiologies that can fall into this category. We do strongly advocate the use of the case definition to standardise results, allow for greater comparison between studies and meta-analysis of data; use of other standards must be interpreted with a degree of caution.

***Nucleic-acid amplification tests***

To address the limitations of conventional microscopy and culture techniques, NAATs have emerged as important tools for rapid and accurate diagnosis of TBM ^44^. A recent meta-analysis evaluating NAATs in TBM reported heterogeneity in results with a pooled sensitivity of 82% against culture and 68% against a clinical reference standard ^68^. This variability, especially in in-house NAATs, is subject to difference in volume of sample, method of extraction, choice of targets used, presence of inhibitors in the sample and lack of optimal reference standard. Traditional NAATs require expensive equipment, stringent operational conditions and technical expertise limiting their use in routine clinical practice in lower-resource, high endemic settings. To circumvent these challenges, loop mediated isothermal amplification (LAMP) assays were developed and can be conveniently carried out under isothermal conditions in an ordinary laboratory water bath or heating block within one hour. Though LAMP has outperformed PCR in an Indian study on TBM ^69^, the assay is still in its infancy and needs further validation. Another method to potentially reduce the overall cost of NAAT would be to utilize magnetic bead assay technology, thus obviating the need of gel electrophoresis system or expensive dyes.

Xpert is a rapid (90 min run-time) fully-automated cartridge-based real-time PCR assay that detects the presence of *M.tb* complex DNA, as well as *rpoB* gene mutations responsible for rifampicin resistance. The pooled sensitivity and specificity of Xpert against culture in 33 studies on TBM, was 71.1% and 98%, respectively ^70^. Xpert has been shown to significantly increase microbiological confirmation of TBM in Uganda over a 6.5-year period but its impact on clinical outcomes in unknown ^71^. Individual studies have also found inferior performance for Xpert compared to multiplex PCR ^72^ or Amplicor assay ^73^ in diagnosing TBM although these results have not been confirmed. The next generation, GeneXpert MTB/Rif Ultra (Ultra) has an 8-fold lower limit of detection than Xpert (16 CFU/ml versus 113 CFU/ml) attributable to a larger chamber allowing double the volume of sample to reach the PCR reaction and two additional DNA probes (IS1081 and IS6110) ^74^. In a Ugandan study of Ultra using cryopreserved CSF, alongside MGIT culture and Xpert, Ultra demonstrated a sensitivity of 95% against a composite microbiological reference and 70% against probable/definite TBM in comparison to 45% and 43%, respectively for each Xpert and culture ^75, 76^. In 2017, Ultra was endorsed by the WHO as the best initial test for TBM and is being rolled out currently worldwide, superseding Xpert ^77^. 

In January 2020, two larger prospective studies evaluating Ultra were published. In the Ugandan study, 204 (96% HIV-positive) adults with suspected meningitis had CSF Xpert Ultra performed. Compared with a reference of definite/probable TBM, test sensitivities were 77% (95% CI 63 – 87%) for Ultra, 56% (95% CI 44 – 70%) for Xpert, and 61% (95% CI 45–76%) for mycobacterial culture ^78^. In this study ‘possible TBM’ cases were not included in the reference standard as this category is non-specific in HIV co-infection due to concomitant infectious and non-infectious brain pathologies associated with advanced immunosuppression. In the second study, Donovan *et al.* employed a different study design and randomised 205 Vietnamese adults (15% HIV co-infected) with meningitis to either Ultra or Xpert testing. Against a reference standard of definite, probable, or possible TBM, test sensitivities were 47% (95%CI, 34 – 60%) for Ultra, 40% (95%CI, 28 – 53%) for Xpert, and 48% (95%CI, 38 – 58%) for mycobacterial culture ^79^. Specificity of Ultra for TBM diagnosis was high in both studies. The sensitivity of Ultra statistically superior to that of Xpert in Uganda but not in a Vietnamese predominantly HIV-negative population. How can we rationalise and interpret these differing results? Firstly, diagnostic tests cannot be expected to perform identically in all settings. Differences in tested CSF volume, CSF processing, HIV co-infection, genetics influencing host response to *M.tb*, and *M.tb* lineages (the number of copies of IS1081 and IS6110 genes varies by lineage) could all contribute to these different results, as could the differences in study design (e.g. head-to-head comparison versus randomizing samples) and smear microscopy sensitivity and reference standards used. Secondly, and most importantly, regardless of the differences in the exact performance of Ultra, the key point is that while Ultra demonstrates some improvement on the performance of Xpert, its negative predictive value is not sufficiently high to exclude TBM when the result is negative.

Another commercial NAAT, the MTBDRplus assay, has been evaluated only in few cases of TBM and needs further validation ^80^. Accurate and rapid detection of drug resistance is another challenge, rifampicin resistance detection by Xpert has imperfect sensitivity (93%) and where detected and ideally requires confirmation by sequencing or culture ^72, 81^. Ultra uses melt curve analysis to improve detection of rifampicin resistance but both are about 95% sensitive ^82, 83^. Ultra will not be able to adequately define rifampin resistance in samples with a low quantity of bacilli (trace category positive) ^75^. In summary NAATs, are a major diagnostic advance but they cannot yet fully replace culture methods. Ultra is too insensitive to rule out TBM, and like Xpert, should be considered as the first test and not the last in TBM diagnosis ^84^. Ultra is an important step in the right direction but the result should be considered in the context of the clinical probability of TBM ^85^.

***CRISPR-MTB and metagenomic next generation sequencing***. Clustered regularly interspaced palindromic repeat (CRISPR) associated proteins (Cas) have the ability to cleave DNA at specific sites and are being used widely in gene-editing and more recently in infectious disease diagnostics. When combined with DNA amplification, the CRISPR system can detect nucleic acid molecules at extremely low abundance. There is one recent report of utilizing the CRISPR system for detection of *M.tb* (CRISPR-MTB). The study included 26 CSF specimens and found CRISPR-MTB to have a sensitivity of 73% compared to 54% for Xpert and 23% for culture against a reference standard of ‘clinical TBM’ as determined by the physician based on clinical presentation imaging and response to TB therapy. The specificity of the test was 98% when tested against 63 non-TB cases. CRISPR-MTB is isothermal and can be performed in under 2 hours using only 500 μl of CSF. CRISPR-MTB remains to be tested against Ultra and requires a higher level of laboratory expertise, resources, and time than the Xpert platform but may be an advance in TB diagnostics if these findings can be confirmed in other settings with more standardized reference standards ^86^.

Metagenomic next generation sequencing (mNGS) is a rapidly developing technology that has proved useful in determining aetiologies for CNS infections that have evaded detection by conventional techniques. Further, mNGS, as opposed to organism-specific molecular tests has the ability to detect *any* low abundance infection with a single test ^87^. A recent small study applied mNGS to stored CSF samples from 23 TBM cases and found a sensitivity of 67% (8/12) against a reference standard of definite TBM, higher than AFB stain (33%, 4/12), PCR (25%, 3/12) and culture (8%, 1/12) ^88^. Paucibacillary conditions such as TBM where the bacillary load may fall below the LOD of commercial NAATs, or where mutations exist around specific PCR primer binding sites may find particular use for mNGS. Targeted enrichment of low abundance genes with Finding Low Abundance Sequences by Hybridization (FLASH), a novel CRISPR-Cas9 technology can increase DNA read abundance by up to 10 ^5^-fold before sequencing occurs ^89^. Combining FLASH and mNGS technologies could improve detection of TB DNA and associated antimicrobial resistance mutations mutations ^90^. A first pilot of FLASH technology in TB demonstrated up to a 100-fold increase in TB read abundance, detection of 6/6 cases of TBM positive with Ultra and detection of an additional case of TBM that had been missed by Xpert, Ultra and MGIT culture ^91^. Here again, large studies need to be performed to better understand this technology’s performance and the cost, laboratory infrastructure, and degree of expertise will need to be improved upon to permit widespread usage.

***Pathogen-based biomarkers***. A urine lateral flow assay (LFA) that detects *M.tb* lipoarabinomannan (TB-LAM), a 17 kDa glycolipid found in the outer cell wall of MTB, has recently been recommended by the World Health Organization for the diagnosis of HIV-associated TB in HIV-positive inpatients (Alere Determine TB-LAM, Abbott, Chicago, USA). The unique characteristic of the test is that its sensitivity increases as CD4 T-cell count falls, with a sensitivity of 56% in those with CD4 <100 cells/ml ^60^. Yet, in CSF, despite some initial optimism related to an autopsy-based study in Uganda, the Alere TB-LAM has shown poor sensitivity on lumbar CSF in Uganda ^92, 93^, along with a larger Zambian study which examined culture positive TBM in Zambia (TB-LAM sensitivity 22% (23/105)) ^92, 94, 95^. The Alere TB-LAM is also limited by its susceptibility to individual reader interpretation of the darkness of the test line compared to the reference card ( Figure 4). A novel LAM assay (Fujifilm SILVAMP TB-LAM, Fujifilm, Japan) is able to detect concentration of LAM at approximately 30-fold lower than Alere TB-LAM due to design differences, including a silver amplification step and gives a result in one hour ^96^. The Fujifilm LAM was recently tested on the CSF of 101 predominantly HIV-positive adults with suspected TBM. FujiLAM sensitivity was 74% (25/34, 95%CI 56-87%) versus definite TBM and 52% (30/58, 95%CI 38-65%) versus probable/definite TBM. The FujiLAM assay may play a role in reducing time to treatment where the Xpert Ultra platform is not immediately accessible or where clinical suspicion of TBM is high despite a negative CSF Ultra result. The sensitivity of FujiLAM was superior to Alere LAM (50% versus 14%, p<0.01 in those tested with both assays) ^97^.

***Clinical prediction rules***

Work is underway to develop a more accurate multivariable clinical prediction rule derived from large international cohorts using individual patient data ^98^. The hope is that a data-driven scoring system will be developed for use in a range of clinical settings by using common, readily available clinical or laboratory parameters to aide in clinical decision making.

## Discussion

In the last five years, the pathogenesis of TBM has been better elucidated, in part thanks to detailed immunological studies on clinical samples preemptively stored during clinical trials. These advances highlight the importance of collecting and storing samples appropriately for future research to maximize scientific outputs, as highlighted in the paper on sampling strategies in this collection. HIV infection is a major predictor of mortality in TBM and advanced HIV infection (CD4 T cell count <150 cell/μl) appears to drive a dysregulated, hyperinflammatory phenotype with very poor outcomes. In HIV-endemic sub-Saharan African settings around 90% of all adult TBM occurs amongst HIV-positive individuals ^71, 95^, often with either untreated advanced HIV or having recently initiated ART - both driving a hyperinflammatory response. In Vietnamese adults with TBM, LTA4H genotype is a strong predictor of mortality though this finding was not duplicated in Indonesia.

Recent insights have shown that neutrophils play a significant role in the immunopathogenesis of TBM, and that both a paucity and an excess of inflammation can be equally damaging in TBM. It has become increasingly clear that a ‘one-size-fits-all’ approach is too simplistic in TBM treatment, as in other infections such as pulmonary TB and sepsis ^99, 100^. The damage-response framework may provide a useful structure for understanding host-pathogen interactions in TBM, illustrating how immune response could be exploited for therapeutic purposes. Additional anti-inflammatory therapy with aspirin ^17, 101^ or more targeted immunotherapy could have a role in persons with an excessive inflammatory response; whilst individuals with an inadequate response might do better without corticosteroid treatment or might even benefit from immunomodulating therapy to boost their immune response ^102^. Future trials of novel specific host-directed therapies are needed and must include immune markers to allow for post-hoc identification of subgroups benefitting from the initiated therapy. Because of the lack of correlation between blood and CSF compartments we advocate inclusion of both blood and CSF markers when studying adjuvant therapies.

The field of TBM diagnosis is rapidly evolving with GeneXpert MTB/Rif Ultra being the most promising test to date for diagnosis of TBM. Ultra is rapid and has potential to confirm more cases of TBM at lower bacillary loads, though whether this will improve outcomes remains to be determined. Most importantly, Ultra does not appear to have adequate predictive value to ‘rule-out’ TBM and so it cannot meet the potential of an ideal TBM diagnostic test to avoid long, toxic TBM therapy in persons without TBM. Novel sequencing technologies hold potential to provide increased understanding of pathogen genomics and behavior and further illuminate host response, which may in turn lead to novel diagnostic and therapeutic targets. Sequencing technologies are increasingly available in TB endemic settings but will need further improvements in affordability and speed in addition to more data on accuracy to unlock their potential as diagnostic tools for TBM. It is now a realistic hope that a test (or set of tests) will one day be available that will be able to confirm or rule out TBM, provide *M.tb* resistance information, and direct clinicians to targeted, adjunctive host-directed therapy within hours.
