# Supplementary material for: Recent Developments in Tuberculous Meningitis Pathogenesis and Diagnostics
Source: Wellcome Open Res. 2021 Jan 28;4:164. Originally published 2019 Oct 31. [Version 3] doi: 10.12688/wellcomeopenres.15506.3 (PMC7739117; doi:10.12688/wellcomeopenres.15506.3)
Supplement: Peer review history

# Peer review: Reviewer response for version 3

**DOI:** 10.21956/wellcomeopenres.18255.r42363

**Published:** 2021-02-04

**Authors:** Joseph Zunt

**Affiliations:** 1 Department of Neurology, Harborview Medical Center, University of Washington, Seattle, WA, USA

No additional comments.

Is the review written in accessible language?

Yes

Are all factual statements correct and adequately supported by citations?

Yes

Are the conclusions drawn appropriate in the context of the current research literature?

Yes

Is the topic of the review discussed comprehensively in the context of the current literature?

Yes

Reviewer Expertise:

Neurology, infectious diseases, epidemiology, global health

I confirm that I have read this submission and believe that I have an appropriate level of expertise to confirm that it is of an acceptable scientific standard.

# Peer review: Reviewer response for version 2

**DOI:** 10.21956/wellcomeopenres.17659.r41505

**Published:** 2020-12-15

**Authors:** Michael J. Griffiths

**Affiliations:** 1 Institute of Infection and Global Health, University of Liverpool, Liverpool, UK

Overall the manuscript represents an excellent review of recent developments in tuberculous meningitis pathogenesis and diagnostics. I agree with changes following the first reviewer’s, Dr Scriven, suggestions. I add two relatively modest comments on the document.

Utilising host biomarkers for TBM diagnostics are an exciting development. In the section on ‘Biomarkers in Children’, the authors describe the results of two studies (by the same group) using different combinations of host CSF biomarkers among children [15,64]. The authors should state what type of host markers (i.e. a mixture of cytokine and soluble mediators) were measured in the studies (using ELISA and Luminex platform). In the follow-up study [64], a relatively small number of patients (n=47) were tested in un-blinded study design. The STARD diagram shows a series of markers (n=69) were measured before the samples were then split into two clinical groups (TBM [definite/probable] n=23 or non-TBM n=24) and later the accuracy of the best combination of markers reported. Given the small size and unblinded design, the results of these studies (particularly the latter) could be summarized further and technical study limitations stated (beyond using CSF).

 In the section on TBM diagnostics- many different types of host biomarkers are now being measured and their diagnostic potential beginning to be assessed in TB, including host transcripts. There are a few publications reporting on host transcripts among TBM patients, including work by the authors (Rohlwink UK *et al*. 2019 ^1^; Hemingway C *et al. *2017 ^2^). While diagnostic accuracy of RNA signatures in TBM have not been assessed in these studies (as stated in Figure 4), there is in an enlarging body of work in pulmonary TB. Reference to some of the larger host transcript studies assessing diagnostic accuracy may be worth mentioning, as these studies highlight the potential strengths/challenges ahead for this approach in TBM (Gupta RK *et al*. 2020 ^3^; Sweeney TE *et al*. 2016 ^4^.

Is the review written in accessible language?

Yes

Are all factual statements correct and adequately supported by citations?

Yes

Are the conclusions drawn appropriate in the context of the current research literature?

Yes

Is the topic of the review discussed comprehensively in the context of the current literature?

Yes

Reviewer Expertise:

Paediatric neurology, CNS infections, diagnostics.

I confirm that I have read this submission and believe that I have an appropriate level of expertise to confirm that it is of an acceptable scientific standard.

## References

[1] : Tuberculous meningitis in children is characterized by compartmentalized immune responses and neural excitotoxicity. *Nature Communications*.2019;10(1) : 10.1038/s41467-019-11783-9 10.1038/s41467-019-11783-9 PMC670415431434901

[2] : Childhood tuberculosis is associated with decreased abundance of T cell gene transcripts and impaired T cell function. *PLoS One*.2017;12(11) : 10.1371/journal.pone.0185973 e0185973 10.1371/journal.pone.0185973 29140996PMC5687722

[3] : Concise whole blood transcriptional signatures for incipient tuberculosis: a systematic review and patient-level pooled meta-analysis. *The Lancet Respiratory Medicine*.2020;8(4) : 10.1016/S2213-2600(19)30282-6 395-406 10.1016/S2213-2600(19)30282-6 31958400PMC7113839

[4] : Genome-wide expression for diagnosis of pulmonary tuberculosis: a multicohort analysis. *The Lancet Respiratory Medicine*.2016;4(3) : 10.1016/S2213-2600(16)00048-5 213-224 10.1016/S2213-2600(16)00048-5 26907218PMC4838193

# Response

**Published:** 2021-01-12

**Authors:** Fiona Cresswell

**Affiliations:** London School of Hygiene & Tropical Medicine, UK

We would like to thank Dr Griffiths for this expert review. 

In response to comment 1.

 Thank you for raising the point requiring further information including the type of host biomarkers and assay methods, and study limitations. For the study (reference 15), we have added the following text: ‘In a study evaluating disease-specific CSF biomarkers of paediatric TBM, a combination of 28 cytokines and soluble mediators were assessed; 27 host biomarkers by Luminex multiplex bead array technology (Bio-Rad Laboratories) and cathelicidin LL-37 concentration by using an enzyme-linked immunosorbent assay (ELISA) kit (USCN Life Science).’

 For the follow-up study (reference 64), we have added the following text: ‘In this study investigating 69 potentially-useful host biomarkers for childhood TBM (23 children with TBM and 24 controls)  comprising a combination of cytokines and soluble mediators in CSF (cathelicidin LL-37 by using an ELISA kit purchased from Elabscience Biotechnology Inc. (catalog #E-EL-H2438) and the rest  by Luminex assay).

 Study limitations were addressed by adding the following text: ‘Limitations of the studies assessing CSF host biomarkers in childhood TBM include relatively small sample size, and therefore few children with confirmed TBM, confirmed meningitis due to other pathogens, and confirmed HIV co-infection. External validation is a necessity in order to generalize the clinical usefulness of the prediction model in an independent group of patients. Despite the potential of CSF-based biosignatures, a further limitation is the invasive nature of CSF collection, and blood or urine-based inflammatory biosignatures require exploration.’

Comment 2. 

 Thank you for raising the point relating to the utility of RNA transcripts as a biomarker for TBM diagnosis. Indeed, a number of recent studies in adults and children have utilised the host transcriptome to better understand the pathogenesis of TBM. Work is underway to use the host transcriptome and develop a machine learning classifier to aid the diagnosis of TBM but this work is unpublished.

 We have added the following text to the paper: “In the field of pulmonary tuberculosis, host RNA transcriptomic signatures have been leveraged to predict incipient and active tuberculosis with moderate short-term (<3 month) sensitivity (41-81%) [Gupta, Lancet Resp Med, 2020]. A whole blood 3-gene signature (GBP5, DUSP3, KLF2) has been shown to accurately differentiate active pulmonary tuberculosis from healthy controls (area under receiver operator curve, AUC 0.90), latent TB infection (AUC 0.88) and other diseases (AUC 0.84) [Sweeney, Lancet Resp Med, 2016]. Whether this 3-gene signature in whole blood has diagnostic value in TBM, whereby the inflammation can be compartmentalised to the CNS, remains to be investigated. An ongoing study in Uganda is adopting a machine learning approach to develop a classifier that categorises patients as TBM or not-TBM based on their CSF RNA signature. The classifier. Four genes predominantly predicted TBM (FTL, NFKBIA, SOD2, GBP5) and the classifier has good sensitivity and specificity (unpublished data).”

 Many thanks again for taking the time to improve the manuscript through these comments.

# Peer review: Reviewer response for version 2

**DOI:** 10.21956/wellcomeopenres.17659.r41544

**Published:** 2020-12-14

**Authors:** Joseph Zunt

**Affiliations:** 1 Department of Neurology, Harborview Medical Center, University of Washington, Seattle, WA, USA

This is a thorough review of current developments in the field of tuberculous meningitis. They included a very nice inclusion of promising new technology, such as CRISPR-MTB and metagenomics. Although not an obligatory recommended change, given the multiple diagnostic assays discussed, I think the reader would find useful a table that listed the various assays with their accompanying sensitivities, specificities and limitations.

Minor recommended edits:

- I agree with Reviewer 1 that the “Rich Focus” was not “refuted” and the editorial change to “potentially challenges” is perhaps not warranted, as there may be more than one pathway to development of meningitis – an event simultaneous with initial infection and a more-delayed event, such as that proposed by Rich and McCordock.
- On page 9, under Pathogen-based diagnostics, the authors start discussing the “potential to score points” without reference to any specific diagnostic criteria – please edit accordingly.
- Page 10; the authors discuss a study in Uganda – It would be helpful to know if the “reference of definite/probable TBM” was made by culture, biopsy or other. Later on this same page, they reference a study of CRISPR-MTB and Xpert that used a standard of “clinical TB” - It would be useful to know what this clinical diagnosis was based on – clinical + CSF findings + neuroimaging findings?

​​​​​​ There were multiple minor grammatical errors that should be corrected:

- P 3. Second to last sentence in the Introduction – “that” should be removed.
- P 5. Second paragraph – “cytokine” should be “cytokines”.
- P6. First paragraph – please remove redundant sentence.
- First sentence under Neuroimaging in pathogenesis studies – “has” should be “have”.
- As mentioned by reviewer 1, comma should follow “anterior cingulate gyrus”.
- P8. Third paragraph – “where” should be “were”.
- P9. Third paragraph – “tests” should be “test”.
- P10. Sixth paragraph – “stanrdards” should be “standards”.
- P11. Second paragraph – “is susceptibility” should be “its susceptibility”.

Is the review written in accessible language?

Yes

Are all factual statements correct and adequately supported by citations?

Yes

Are the conclusions drawn appropriate in the context of the current research literature?

Yes

Is the topic of the review discussed comprehensively in the context of the current literature?

Yes

Reviewer Expertise:

Neurology, infectious diseases, epidemiology, global health

I confirm that I have read this submission and believe that I have an appropriate level of expertise to confirm that it is of an acceptable scientific standard.

# Response

**Published:** 2021-01-12

**Authors:** Fiona Cresswell

**Affiliations:** London School of Hygiene & Tropical Medicine, UK

We sincerely thank Dr Zunt for his expert review and suggestions.

 In response to major comment:

 We have added a table summarising the characteristics of CSF tests for TBM and added a couple of lines about CSF FujiLAM following a recent publication relating to this assay in TBM.  

 In response to minor comments:

 1. We have edited the text to reflect the fact that there may be more than one route to TBM disease.  

 2. We have removed this text about the points relating to the reference standard in the interest of brevity.

 3. In the Ugandan study MGIT culture and Xpert were used alongside Ultra, this detail has been added to the paper. In the CRISPR study “clinical TB” was as determined by the physician based on clinical presentation imaging and response to TB therapy. This has been added to the paper.

 We have rectified the multiple typos spotted. 

 Many thanks for your suggestions which have improved the manuscript.

# Peer review: Reviewer response for version 2

**DOI:** 10.21956/wellcomeopenres.17659.r39409

**Published:** 2020-09-02

**Authors:** James E. Scriven

**Affiliations:** 1 University of Birmingham, Birmingham, UK

Thank you for taking on board my comments and making the changes and clarifications detailed in your response. I am happy that the sections previously highlighted have now been explained fully.

 The only think I have noticed on the re-reading is the second sentence in the **Brain Injury Markers** section (copied in below) doesn't quite make sense.Could the authors please review this. I think there may be some punctuation (and possibly words) missing?  

 ".....In lumbar CSF of children with TBM, S100B and NSE (structural proteins of the CNS, and biomarkers of CNS tissue damage) at disease onset were associated with poor outcome, as was highest concentration overall and an increasing profile over time in S100B, NSE, and GFAP neuromarker concentrations increased over time in those who died (whilst inflammatory markers decreased), and were overall highest in those with cerebral infarction ^32^."

Is the review written in accessible language?

Yes

Are all factual statements correct and adequately supported by citations?

Yes

Are the conclusions drawn appropriate in the context of the current research literature?

Yes

Is the topic of the review discussed comprehensively in the context of the current literature?

Yes

Reviewer Expertise:

Infectious Diseases; CNS infections; immunology

I confirm that I have read this submission and believe that I have an appropriate level of expertise to confirm that it is of an acceptable scientific standard.

# Peer review: Reviewer response for version 1

**DOI:** 10.21956/wellcomeopenres.16971.r36984

**Published:** 2020-01-13

**Authors:** James E. Scriven

**Affiliations:** 1 University of Birmingham, Birmingham, UK

Thank you for asking me to review this manuscript.

**General comments**

 This is a comprehensive review of TB meningitis pathogenesis and diagnostics with the stated aim of concentrating on the most recent developments (last 5 years).

 The authors should be commended on providing a detailed review. They have included the vast majority of recently published studies but should also include the Vietnamese study published in Lancet ID this month by Donovan et al that compares Xpert against XpertUltra in TBM (and compare it to their recently published study in HIV-TBM patients). ^1^

 My main criticism of the review relates to the section on **TBM pathogenesis** which requires further work to improve understanding for the reader. There are several sections where the authors have listed a series of findings from separate immunological studies but do not introduce the concept fully to the reader and do not draw the findings together at the end of each section to provide an overall interpretation or explanation for the reader. Some further explanation around these concepts would significantly improve the review.

 This is particularly true for the section covering host genetic factors and the influence of LTA4H genotype on host response and outcome. This is a really important and fascinating piece of science but for readers not already familiar with this concept this section is confusing. The authors should provide further explanation, particularly regarding the differing inflammatory states associated with the three genotypes, and why it is important. They should consider including discussion and reference of the original paper by Tobin et al (Cell 2012). ^2^

**Specific comments:**

**Introduction**

- The GeneXpert test performances should be referenced. I think there is a typo on line 7 ("traditional" should be "traditionally")?

**TBM pathogenesis - Dissemination to the CNS**

- The use of the word **refute **with regard to the Rich focus theory of TBM pathogenesis could be regarded as a bit strong. Do the authors think that the references provided fully support this statement?

**TBM pathogenesis - Host immune response to TB infection in the CNS**

- The first two sentences of the first paragraph in this section should probably contain a reference.
- In the second paragraph, the association between death and lower CSF leukocyte count should include the p=value.
- Further explanation/interpretation of the lipid mediator findings and flow cytometery findings would help improve understanding of this section.
- The authors should also consider discussing the Vietnamese phase II trial of Aspirin by Mai et al (Ref 94), further in this section. They very briefly mention it in the Discussion section but a more detailed discussion is probably warranted and it may be appropriate to do so here.

**TBM pathogenesis - Host genetic and metabolic factors. **

- As mentioned above, for readers not familiar with the original studies this section discussing LTA4H genotypes is difficult to understand as it is currently written. It would benefit from re-wording and further explanation, particularly a sentence or two to introduce the concept at the beginning of the paragraph and a precise explanation of the effects of LTA4H genotype on inflammation, steroid responsiveness and outcome (with reference to the original paper, Tobin et al. Cell.  2012). ^2^
- The authors should also consider adding a couple sentences to the section on tryptophan to provide some additional information about this study and providing a more comprehensive explanation for the association between low levels and better survival.

**Figure 2. **

- The figure should make it clear that the patients in A and B have all received steroids. It would also aid understanding if the figure legend also contained the p=values with respect to the differing outcomes quoted for graph A and B. The final sentence looks like it might contain a typo:
*"In a recessive model, **TT genotype versus CT/TT **combined had HR 0.81 (95% CI 0.41-1.62, p = .550) in severe and 0.31 (95% CI 0.04-2.25, p = .156) in milder disease." *
  I think the comparison stated in the model should be be **TT vs CT/CC**?

**TBM pathogenesis - HIV co-infection and IRIS**

- In the first paragraph (line 9, copied below), a comparison in neutrophil counts and mortality is presented between patients with a CD4 count >150, CD4 <150 and HIV negative.
*"PLWHIV with CD4+ T-cell counts <150 cells/μL showed higher median CSF neutrophil percentage (25%), cytokine concentrations and 9-month mortality (44%) than those with a CD4+ T-cell count ≥150 cells/μL (neutrophils 10%; **P=.021**, mortality 13%) and patients without HIV infection (neutrophils 5%; **P<.0001,** mortality 18%). These findings, amongst others, suggest a role for neutrophils in the immunopathogenesis of HIV-associated TBM."*
  However, there are only two p values included and it is not clear whether they refer to neutrophils or mortality. Could the authors please alter this to make it a little clearer (and if appropriate add the two additional p-values).

**TBM pathogenesis - Pathogenesis of TBM in childhood**

- I don't think this section in its current form particularly adds to the review and there is no new data. I would suggest this section is either expanded (to further discuss the differences between adult and paediatric patients with TBM and the pathological processes underlying these differences) or dropped. If the authors do decide to drop it they could consider amalgamating the statements about Donald and Schoeman's TBM and milary TB findings into the earlier section: "Dissemination to the central nervous system".

**TBM pathogenesis - Brain injury markers**

- This paragraph would also benefit from further explanation so that the reader can appreciate the importance of the findings. Further detail about the brain injury markers S100B and NSE would be helpful (particularly whether they are used in other neurological conditions). Further text is also required to explain what the CSF transcriptomic signature means.

**TBM pathogenesis - Neuroimaging in pathogenesis studies**

- Is there a comma missing here?: "anterior cingulate gyrus parahippocampal gyrus and globus pallidus".
- The novel imaging studies reporting structural damage in cortical and subcortical areas are interesting. Perhaps the authors could expand slightly to suggest the potential implications of this?

**TBM pathogenesis - Host pathogen interactions. **

- This section discusses the damage response framework concept and applies it to TBM. I am not sure why the first sentence includes data on global latent TB prevalence - it doesn't seem directly relevant to this section.
- It may improve the review if the authors expanded this section slightly and used it as a summary to draw together all the immunological findings discussed above. Particularly to draw attention to the importance of neutrophils.

**TBM diagnostics**

- This section provides a good summary of host-based and pathogen-based diagnostics and reads well.  
- With regard to the host-based biomarkers, some of the pooled sensitivities appear promising but have drawbacks. It would be useful if the authors could provide a concluding comment at the end of the section discussing whether there is any future in these modalities (e.g. are any of those mentioned being taken forward into further trials? Are there any plans to introduce any of these tests?).
- Regarding the performance of XpertUltra in TBM. The authors have mentioned their prospective study examining its performance in Uganda (reference 70). They should emphasize that these results were in patients with HIV infection. They should also mention the similar study from Vietnam published alongside theirs this month in Lancet ID by Donovan et al which reports lower sensitivities and no significant improvement on Xpert. They should discuss why this might be.

Is the review written in accessible language?

Yes

Are all factual statements correct and adequately supported by citations?

Yes

Are the conclusions drawn appropriate in the context of the current research literature?

Yes

Is the topic of the review discussed comprehensively in the context of the current literature?

Yes

Reviewer Expertise:

Infectious Diseases; CNS infections; immunology

I confirm that I have read this submission and believe that I have an appropriate level of expertise to confirm that it is of an acceptable scientific standard, however I have significant reservations, as outlined above.

## References

[1] : Xpert MTB/RIF Ultra versus Xpert MTB/RIF for the diagnosis of tuberculous meningitis: a prospective, randomised, diagnostic accuracy study. *The Lancet Infectious Diseases*.2020; 10.1016/S1473-3099(19)30649-8 10.1016/S1473-3099(19)30649-8 PMC704508831924551

[2] : Host genotype-specific therapies can optimize the inflammatory response to mycobacterial infections. *Cell*.2012;148(3) : 10.1016/j.cell.2011.12.023 434-46 10.1016/j.cell.2011.12.023 22304914PMC3433720

# Response

**Published:** 2020-06-21

**Authors:** Fiona Cresswell

**Affiliations:** London School of Hygiene & Tropical Medicine, UK

We thank the reviewer for taking the time to provide this very considered and detailed review. We have taken Dr Scriven's comments on board, modified the paper accordingly, and we hope the article has been strengthened as a result. In response to Dr Scriven's comments we have made the following changes:

- Added a paragraph relating to the two recent studies on Xpert MTB/RifUltra as suggested. 
- We have added a reference to the introduction line 7 as suggested 
- The statement regarding 'refuting' the Rich focus has been made less conclusive
- P value added to the paragraph about CSF leucocytes 
- Further developed the sections relating to pathogenesis:
  - expanded section on aspirin study and lipid mediators
  - expanded background on LTA4H
  - expanded evidence around brain injury markers
- We have not added further information on tryptophan in the interest of word limit as there is already a full paragraph on this topic
- The section on pathogenesis of TBM in childhood has been removed as suggested. Donald and Schoeman’s TBM and miliary TB findings are now included in the "Dissemination to the central nervous system" section
- We have added a summary statement on TBM biomarkers explaining that despite extensive research on this topic there are no currently biomarkers with adequate performance to be commercially viable in TBM diagnostics.
- Typos and grammatical errors have been corrected. Many thanks! 

We are very grateful for Dr Scriven's expertise and hope he is satisfied with the updated article.
